# Ribosome profiling of the retrovirus murine leukemia virus

**DOI:** 10.1186/s12977-018-0394-5

**Published:** 2018-01-22

**Authors:** Nerea Irigoyen, Adam M. Dinan, Ian Brierley, Andrew E. Firth

**Affiliations:** Division of Virology, Department of Pathology, University of Cambridge, Tennis Court Rd, Cambridge, CB2 1QP UK

**Keywords:** Retrovirus, Ribosome profiling, RNASeq, Viral translation, Murine leukemia virus, Upstream ORF

## Abstract

**Background:**

The retrovirus murine leukemia virus (MuLV) has an 8.3 kb RNA genome with a simple 5′-*gag*-*pol*-*env*-3′ architecture. Translation of the *pol* gene is dependent upon readthrough of the *gag* UAG stop codon; whereas the *env* gene is translated from spliced mRNA transcripts. Here, we report the first high resolution analysis of retrovirus gene expression through tandem ribosome profiling (RiboSeq) and RNA sequencing (RNASeq) of MuLV-infected cells.

**Results:**

Ribosome profiling of MuLV-infected cells was performed, using the translational inhibitors harringtonine and cycloheximide to distinguish initiating and elongating ribosomes, respectively. Meta-analyses of host cell gene expression demonstrated that the RiboSeq datasets specifically captured the footprints of translating ribosomes at high resolution. Direct measurement of ribosomal occupancy of the MuLV genomic RNA indicated that ~ 7% of ribosomes undergo *gag* stop codon readthrough to access the *pol* gene. Initiation of translation was found to occur at several additional sites within the 5′ leaders of the *gag* and *env* transcripts, upstream of their respective annotated start codons.

**Conclusions:**

These experiments reveal the existence of a number of previously uncharacterised, ribosomally occupied open reading frames within the MuLV genome, with possible regulatory consequences. In addition, we provide the first direct measurements of stop codon readthrough efficiency during cellular infection.

**Electronic supplementary material:**

The online version of this article (10.1186/s12977-018-0394-5) contains supplementary material, which is available to authorized users.

## Background

**Fig. 1:**
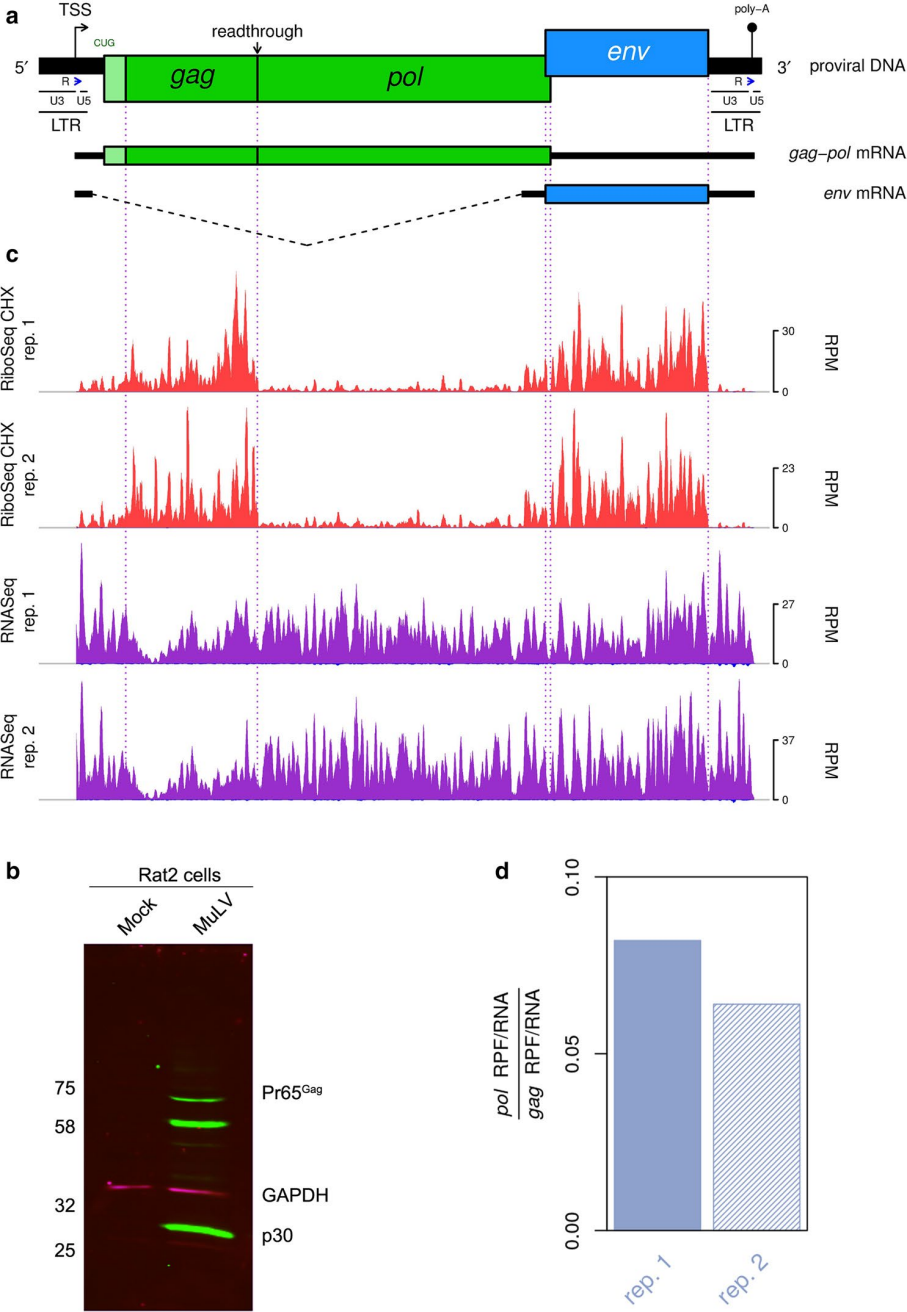
MuLV RNA synthesis and translation. **a** Map of the Moloney MuLV proviral DNA, showing the locations of the untranslated 3′ (U3), R, and U5 repeats within the LTRs, and the *gag*, *pol* and *env* coding regions. U3 houses the viral promoter region, while the transcription start site (TSS) corresponds to the first nucleotide of the R region. The poly-adenylation (poly-A) signal also occurs within R. Translation of *pol* is dependent upon readthrough of the *gag* gene termination codon. Two distinct mRNA species are produced from the provirus template, with the *gag*-*pol* transcript being unspliced and the *env* transcript being spliced at the indicated positions. **b** Rat2 cells were infected with MuLV and at 96 h p.i. cell lysates were prepared, separated by 10% SDS-PAGE and immunoblotted using a polyclonal anti-capsid (p30) serum. Molecular weights (MW; in kDa) are indicated on the left. GAPDH was used as a loading control. Viral proteins were detected with a green fluorescent secondary antibody, and GAPDH was detected with a red fluorescent secondary antibody. **c** RiboSeq CHX (red) and RNASeq (purple) densities in reads per million mapped reads (RPM), smoothed with a 31-nt sliding window. Negative-sense reads are shown in dark blue below the horizontal axes. **d** Bar plot showing the density of RPFs mapped to the *pol* gene relative to the *gag* gene, normalised by the equivalent density of RNASeq reads in these regions. The ratio of *pol* to *gag* translation is approximately 0.07, on average; indicating that ~ 7% of ribosomes undergo stop codon readthrough to translate *pol*

Murine leukemia virus (MuLV), the prototype *Gammaretrovirus*, has long served as a model for the study of retrovirus molecular biology. The viral genomic RNA (gRNA) is approximately 8 kb in length; encoding the *gag*, *pol*, and *env* genes in the 5′–3′ direction (Fig. 1a). Replication of the gRNA depends upon the binding of a host-derived tRNA, which primes the synthesis of negative-sense cDNA intermediates that are subsequently converted to dsDNA harbouring long terminal repeats (LTRs) at the 5′ and 3′ termini (Fig. 1a) [1]. The LTRs contain the sequences required for integration of the virus into host chromosomal DNA, as well as regulatory elements that promote RNA polymerase II-dependent transcription of viral RNA from the integrated provirus. These RNA transcripts may be spliced to give rise to mRNAs encoding the envelope (Env) glycoproteins (Fig. 1a); or they may remain unspliced, in which case they can either be utilised for the translation of the Gag and Gag-Pol polyproteins (Fig. 1a), or packaged into newly synthesised virions as non-covalently associated gRNA dimers [2, 3].

The development of advanced RNA sequencing methodologies in recent years has made it possible to monitor viral gene expression at single nucleotide resolution [4–6]. We carried out sequencing of total RNA (RNASeq) and sequencing of ribosome-protected RNA fragments (RPFs) (RiboSeq)—also known as ribosome profiling—of MuLV-infected Rat2 cells (a rodent-derived cell line). These data allowed us to delineate the transcription and translation of MuLV RNA in unprecedented detail.

## Results

### Ribosome profiling and RNA sequencing of MuLV-infected cells

In order to produce infectious virions, the full-length MuLV Moloney strain proviral clone pNCA [7] (GenBank accession number: NC_001501) was transfected into 293T monolayers at 40% confluence. Cells were harvested at 72 h post-transfection and tissue culture supernatant used to infect in duplicate fresh Rat2 (*Rattus norvegicus*) monolayers at 10% confluence [8]. Cells were harvested at 96 h post-infection (p.i.) and treated with cycloheximide (CHX). In an additional experiment, cells were treated with harringtonine (HAR) followed by CHX. Virus infection was confirmed by western blot for Pr65^Gag^ (Gag) using a polyclonal antibody for the capsid protein p30 (Fig. 1b).

HAR is utilised specifically for the probing of initiation sites due to its capacity to stall initiating ribosomes [9]. RiboSeq CHX, RiboSeq HAR and RNASeq libraries were prepared for each biological sample and deep sequenced. A meta-analysis of reads mapping to host RefSeq mRNA transcripts showed excellent phasing of the RiboSeq libraries for both biological repeats (Additional file 1: Fig. S1). Ribosomal occupancy of these transcripts in the HAR-treated sample was strongly enriched at known initiation codons, whereas the coverage in the CHX-only samples was more uniformly distributed across coding regions (Additional file 1: Fig. S1).

Figure 1c shows the normalised RiboSeq CHX (red) and RNASeq (purple) positive-sense coverage of the MuLV RNA. For all libraries, negative-sense mapping reads (dark blue) were present in much lower quantities than positive-sense mapping reads. The RiboSeq read density was considerably higher in the *gag* and *env* coding regions compared with *pol*. This is expected, given that *pol* translation is dependent upon a proportion of ribosomes undergoing stop codon readthrough (see below). The lengths of RPFs mapping to both viral and host RNA showed a sharp peak at 29 nt, consistent with the size of mRNA fragments protected by translating eukaryotic ribosomes from digestion by RNase I [10] (Additional file 1: Fig. S2). In contrast, the length distribution of virus-mapped RNASeq reads was much broader (Additional file 1: Fig. S2), as it is largely determined by the region excised from the gel during the gel size selection step of RNASeq library preparation (corresponding to approximately 28–34 nt).

### *Gag* gene stop codon readthrough efficiency

Translation of the *pol* ORF requires readthrough of the *gag* amber (UAG) termination codon, which is decoded by glutamine (CAG) tRNA, through wobble base-pairing in the 3′ position of the anticodon [11]. Suppression of termination is promoted by a 63-nt readthrough signal located downstream of the *gag* UAG, consisting of an 8-nt purine-rich spacer sequence followed by an RNA pseudoknot structure [12–14]. To estimate the efficiency of readthrough, we measured the density (as reads per kilobase per million mapped reads; RPKM) of RPFs mapping to the *gag* and *pol* coding regions, and normalised these values by the equivalent RNASeq read densities to at least partly control for PCR and ligation biases. As can be seen in Fig. 1d, we found that 6–8% of ribosomes proceed beyond the *gag* UAG codon and translate *pol*. These data constitute the first direct measurements of the relative ribosomal occupancy of *gag* and *pol* during MuLV infection, and are in close agreement with previous in vitro reporter assays that have been used to estimate the readthrough efficiency [8]. The efficiency of *gag* stop codon readthrough is therefore ~ 100 fold above the background level (i.e. that observed in the absence of specific stimulatory sequences [15]). This regulatory strategy appears to allow the virus to tightly and reliably control the stoichiometric ratio of the associated protein products [8].

### Translational initiation in the 5′ untranslated region

The MuLV Gag polyprotein can be synthesised as either of two distinct isoforms, which are 65 and 75 kDa in size, respectively [16]. The latter of these (“Glyco-Gag”) is N-terminally extended relative to the former, and contains a signal peptide that directs the polyprotein to the rough endoplasmic reticulum, where it undergoes glycosylation, before being trafficked through the Golgi apparatus and displayed on the cell surface [17, 18]. Glyco-Gag appears to function in maintaining viral capsid integrity and in facilitating viral evasion of the host innate immune response [17, 19, 20].

**Fig. 2:**
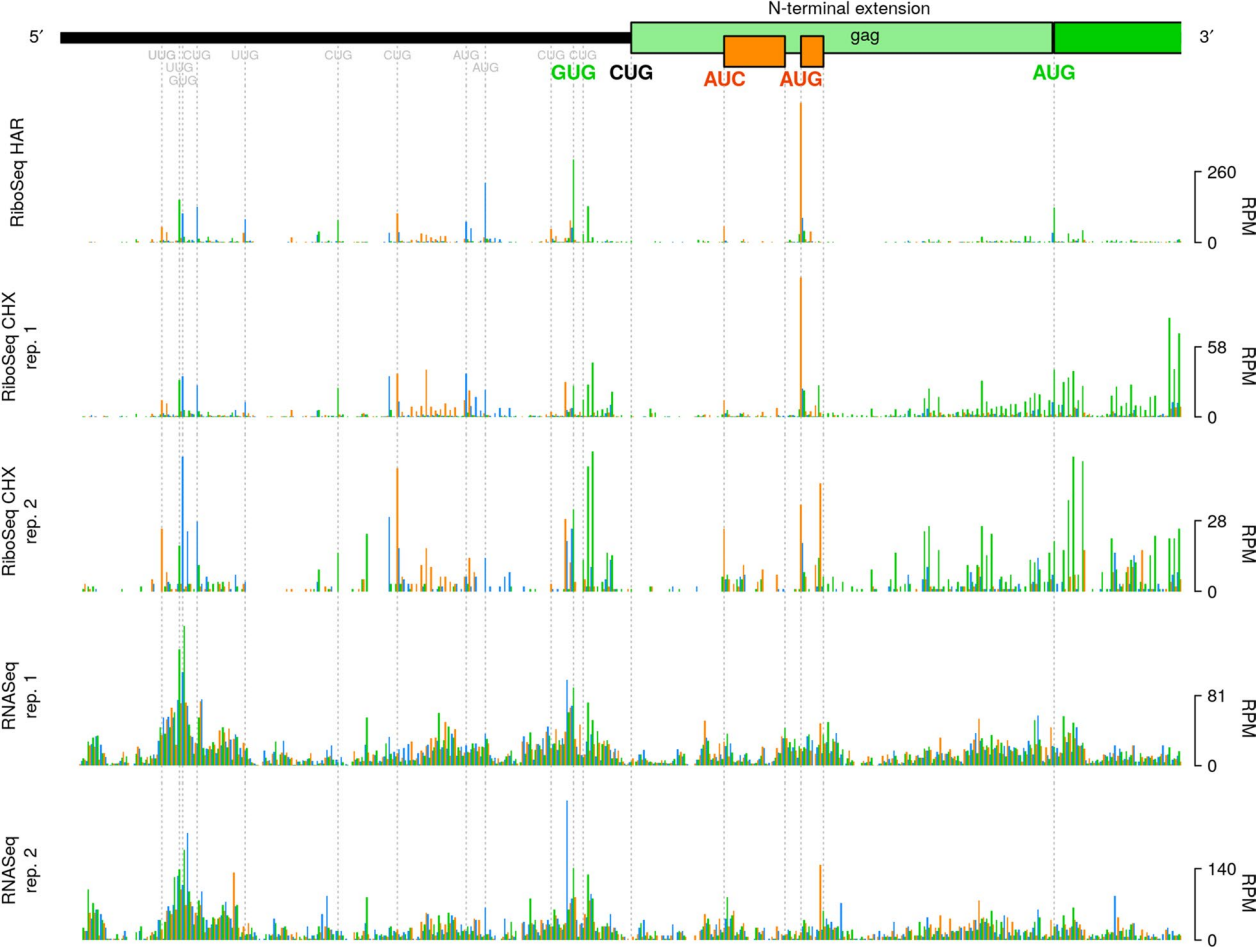
Coverage of the 5′ region of the MuLV genomic RNA. Histograms show the number of 5′ read ends (with + 12 nt offset) mapped to viral RNA in RiboSeq HAR, RiboSeq CHX and RNASeq libraries generated from Rat2 cell infections, in reads per million (RPM). The light green rectangle represents the N-terminal extension of the Glyco-Gag-encoding sequence described in [16], while the darker green rectangle represents the standard *gag* coding sequence. The GUG initiation codon highlighted in green is in-frame with the *gag* N-terminal extension. Intervening AUC and AUG codons at which initiation was observed are shown in orange. Potential additional upstream initiation codons with coverage in the RiboSeq HAR library are shown in grey. Green, orange and blue bars correspond to reads mapping in phases 0, + 1 and + 2, respectively, relative to the *gag* ORF

We found that the average RiboSeq CHX density (by counting reads that are in-phase with either ORF and normalizing by RNASeq) in the N-terminal extension region was 6.7% of that within the standard *gag* ORF, consistent with previous reports [21]. Previously, it has been reported that initiation of Glyco-Gag synthesis begins at a non-canonical (CUG) initiation codon located at nt 357–359 of the MuLV genome [16]. This codon occurs within a favourable context (accCUGg) [16] (in mammalian cells, the presence of an A residue at position − 3, or G residues at − 3 and + 4—relative to the first nucleotide of a potential start codon—is favourable for initiation [22, 23]). Surprisingly, however, our RiboSeq HAR library reveals very low coverage at this CUG codon (Fig. 2—light green rectangle represents the N-terminal extension of *gag*, as per [16]). It is possible that technical biases—for example nuclease bias, PCR bias, ligation bias, or some combination of these—resulting from variations in local nucleotide composition might contribute towards the low coverage observed at this CUG. Supporting this possibility, RNASeq density was also particularly low at this position.

Conversely, we found clear evidence in our RiboSeq HAR library for initiation of translation at an in-frame GUG codon located 12 codons upstream (nt 321–323), which also occurs within a strong initiation context (gugGUGg) (Fig. 2—GUG codon highlighted in green). Translation may proceed contiguously from this codon through the *gag* ORF; thus, presumably, giving rise to an alternative Glyco-Gag isoform which is N-terminally extended by a further 12 amino acids (MELTSSEHPAAT, assuming GUG is decoded by Met-tRNA_i_) relative to the canonical Glyco-Gag. The GUG codon is present in some but not all MuLV strains, and is not well conserved among other gammaretroviral lineages for which sequence data are currently available (Additional file 1: Fig. S3—highlighted in green), although some sequences have alternative nearby potential non-AUG initiation sites. Note that a 77 kDa Glyco-Gag species of unknown origin has been described previously [16, 24] and might represent an N-terminally extended Glyco-Gag. While based on previous work [16] the CUG is probably the main phylogenetically conserved Glyco-Gag initiation site, our results indicate that other nearby initiation sites may contribute additional Glyco-Gag variants.

Within the region encoding the N-terminal extension of Glyco-Gag, we also detected ribosomal initiation at AUC and AUG codons (initiation contexts: aaaAUCc and gauAUGu) associated with 12-codon and 4-codon upstream ORF (uORFs), respectively; both of which are in the + 1 reading frame relative to *gag* (Fig. 2—orange ORFs). Because of the short length of these uORFs, it is possible that the 40S subunits of ribosomes which initiate at these positions may remain associated with the mRNA transcript post-termination, resume scanning, and proceed to reinitiate on the *gag* AUG [25]. The genomes of other gammaretroviruses generally lack AUG codons in the *gag* N-terminal extension region. A notable exception is feline leukemia virus; however, in this case, the AUG is in-frame with the *gag* ORF, without any intervening stop codons, and hence is likely to serve as the Glyco-Gag translational initiation site [26]. Initiating ribosomes were observed at a number of other upstream sites (Fig. 2); upstream ORFs in other retroviruses have been found to modulate viral gene expression and replication [27, 28].

### Translation of the spliced *env* mRNA transcript

**Fig. 3:**
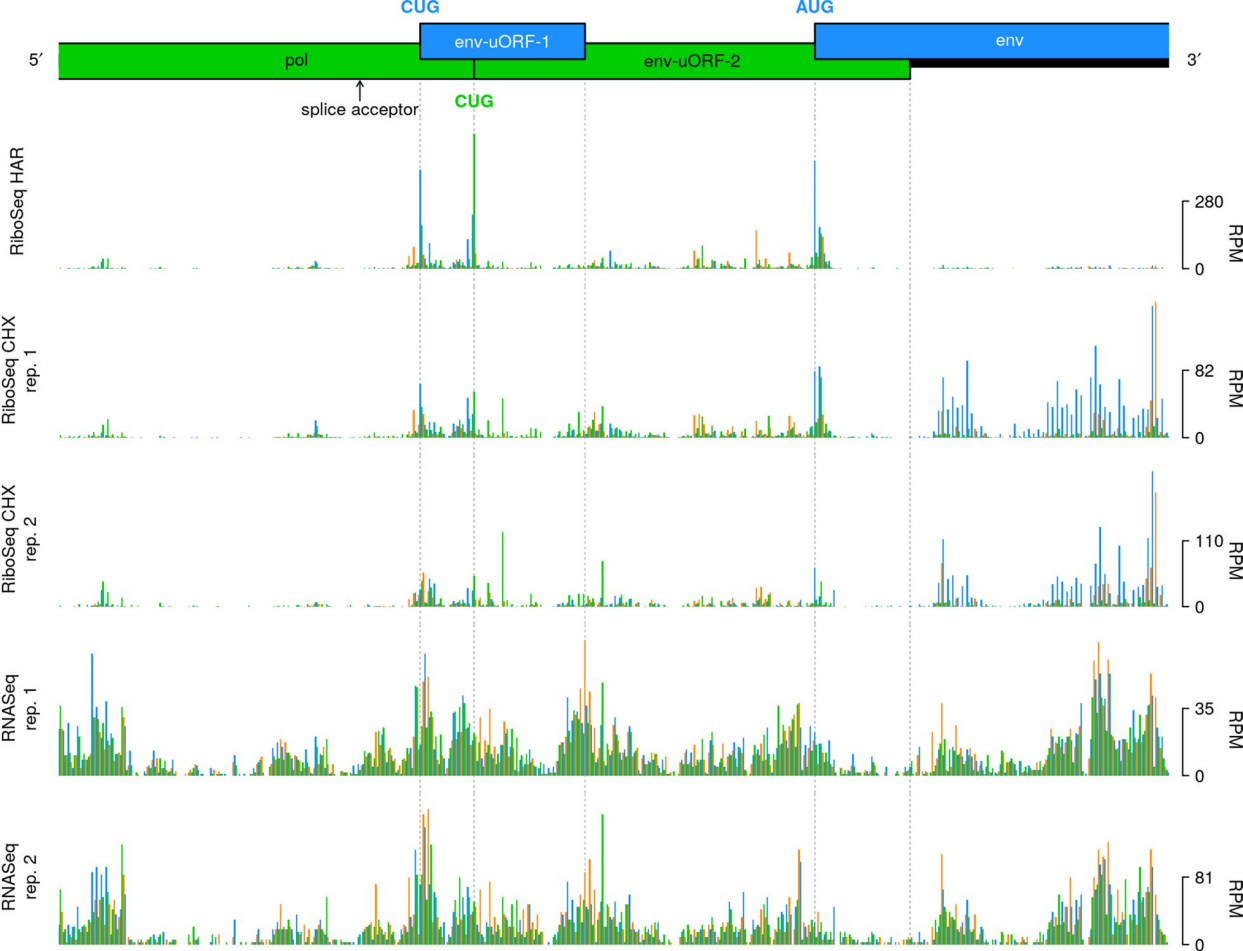
Coverage of the region surrounding the MuLV *env* gene splice acceptor. Bar plots show the number of 5′ read ends (with + 12 nt offset) mapped to viral RNA in RiboSeq HAR, RiboSeq CHX and RNASeq libraries generated from Rat2 cell infections, in reads per million (RPM). Blue, green and orange bars correspond to reads mapping in phases 0, + 1 and + 2, respectively, relative to the *env* ORF. Translation of env-uORF-1 and env-uORF-2 is initiated from CUG codons that coincide with large peaks in RiboSeq HAR data

The *env* gene of MuLV is translated from a spliced mRNA transcript, for which the major splice donor and splice acceptor sites are located at nt 206 and nt 5490 of the full-length gRNA, respectively [29]. Accordingly, the density of mapped RiboSeq reads increased substantially downstream of the *env* splice acceptor, relative to the *pol*-encoding region immediately upstream (Fig. 1c). Two clear ribosomal initiation peaks were observed at two CUG codons located at nucleotides 249 and 215 upstream of the AUG *env* initiation codon (Fig. 3). The first CUG is found within a strong initiation context (aguCUGg), and is associated with a 34-codon uORF (“env-uORF-1"). The second occurs within a medium to poor initiation context (caaCUGg) and is associated with a 91-codon uORF (“env-uORF-2”) that is in-frame with the *pol* gene and encodes the C-terminal tail of the MuLV integrase (Fig. 3). In agreement with the HAR initiation profiling data, the RiboSeq CHX density (normalized to RNASeq density) within env-uORF-2 was four-fold higher, on average, than that in the remainder of the *pol* gene. An alternative splice form, SD′, has been identified that uses a splice donor in the middle of *gag* (nt 1596) and the canonical *env* splice acceptor; SD′ encodes a product comprising the N-terminal part of Gag fused to the C-terminal end of Pol [30, 31]. However, the excess ribosome density at the 3′ end of *pol* clearly starts at the env-uORF-1 and env-uORF-2 CUG initiation codons, not at the SD′/*env* splice acceptor site (Fig. 3), thus indicating that the bulk of translation in env-uORF-2 comes from initiation on the *env* transcript rather than translation of the *gag*-*pol* fusion ORF on the SD′ transcript. Interestingly, the C-terminal region of the integrase that would be encoded by env-uORF-2 is known to interact with the bromodomain and extra-terminal (BET) protein family members, which in turn interact with acetylated histone tails, thus facilitating integration of the proviral genome at actively transcribed regions of the host chromosome [32, 33].

To assess the conservation of these two CUG codons, and the potential for translation of the integrase C-terminal region as an independent protein in other gammaretroviral lineages, we generated a codon-based alignment of *pol* genes (Additional file 1: Fig. S4). The CUG initiation codon of env-uORF-1 (Additional file 1: Fig. S4—highlighted in green) is conserved (or, in two cases, substituted with GUG or AUG) in most but not all lineages, and—where it is present—the context is always favourable for initiation: either with an A at − 3, or with a G at − 3 and a G at + 4. However, the lengths of the associated ORFs vary across lineages—ranging from 8 to 35 codons—and the corresponding amino acid sequences are also variable, suggesting that env-uORF-1 is unlikely to encode a functional peptide; nevertheless, its presence may serve to modulate *env* translation.

The CUG codon of env-uORF-2 is not conserved in other gammaretroviral lineages (Additional file 1: Fig. S4—highlighted in purple). However, the genomes of most lineages harbour multiple potential alternative initiation sites that are in-frame with the *pol* gene and within the *env* transcript leader (Additional file 1: Fig. S4—codons highlighted in cyan). Most of these possible alternative initiation codons have contexts which are at least as strong as that of the MuLV env-uORF-2 CUG. Hence, it is possible that distinct initiation sites are used for the translation of the integrase C-terminal domain. It should also be noted that the Rhinolophus ferrumequinum retrovirus *pol* gene is split into two separate coding regions; the latter of which encompasses the C-terminal tail of the integrase [34] and is included in the given alignment.

## Conclusions

This study constitutes the highest resolution analysis of the transcriptional and translational landscapes of a retrovirus generated to date. Our data indicate the existence of new translation initiation sites, new translated short ORFs and the first measurement of the *gag*-*pol* stop codon readthrough efficiency in the context of the full-length virus genome during infection. These data may contribute towards an improved understanding of the molecular biology of this important model retrovirus.

## Methods

### Virus assays

MuLV replication was assessed by transfection of the proviral clone and subsequent infection of Rat2 cells with virions released from transfected cells. 293T monolayers at 40% confluence were prepared in six-well plates and transfected with pNCA. At 72 h p.t. tissue culture supernatants were filtered through a 0.45-µm-pore-size filter prior to infection of fresh monolayers. Filtered supernatant (3 ml) from transfected cells was added directly to 10-cm plates of Rat2 monolayers at 10% confluence and incubated for 1 h at 37 °C, and the volume was adjusted to 10 ml with fresh medium (DMEM with 2% FCS) in the presence of 30 µg/µl of DEAE-dextran hydrochloride (Sigma). Cells were incubated at 37 °C and 10% CO_2_ for 4 days.

### Immunoblotting

Proteins were separated by 10% SDS-PAGE and transferred to nitrocellulose membranes. These were blocked for 30–60 min with 5% powdered milk (Marvel) in PBST (137 mM NaCl, 2.7 mM KCl, 10 mM Na_2_HPO_4_, 1.5 mM KH_2_PO_4_, pH 6.7, and 0.1% Tween 20) and probed with polyclonal rabbit anti-MuLV p30 (ab130757; Abcam) (1:2000 in Marvel-PBST) and monoclonal mouse anti-GAPDH (G8795; Sigma-Aldrich) (1:20,000 in Marvel-PBST). Membranes were incubated in the dark with an IRDye-conjugated secondary antibody in PBST (IRDye 800CW donkey anti-rabbit IgG_H+L_ and IRDye 680RD goat anti-mouse IgM [µ chain specific]).

### Drug treatment and lysis

At 96 h p.i. cells were treated with CHX (Sigma-Aldrich; to 100 µg/ml; 2 min), or HAR (LKT laboratories; 2 µg/ml, 3 min) then CHX (to 100 µg/ml; 2 min). Cells were rinsed with 5 ml of ice-cold PBS, the dishes were submerged in a reservoir of liquid nitrogen for 10 s and then transferred to dry ice and 400 µl of lysis buffer [20 mM Tris–HCl pH 7.5, 150 mM NaCl, 5 mM MgCl_2_, 1 mM DTT, 1% Triton X-100, 100 µg/ml cycloheximide and 25 U/ml TURBO DNase (Life Technologies)] dripped onto the cells. Cells were scraped, collected and triturated with a 26-G needle ten times. Lysates were clarified by centrifugation for 20 min at 13,000*g* at 4 °C.

### Ribosomal profiling and RNASeq

Cell lysates were subjected to RiboSeq and RNASeq as previously described [35]. The methodologies employed were based on the original protocols of Ingolia and colleagues [36, 37], except ribosomal RNA contamination was removed by treatment with duplex-specific nuclease (DSN) and library amplicons were constructed using a small RNA cloning strategy adapted to Illumina smallRNA v2 to allow multiplexing.

### Computational analysis of RiboSeq and RNASeq data

Adaptor sequences were trimmed using the FASTX-Toolkit (hannonlab.cshl.edu/fastx_toolkit/) and reads shorter than 25 nt following adaptor trimming were discarded. Trimmed reads were then mapped to host (*Rattus norvegicus*) and virus RNA using bowtie version 1 [38], with parameters-v 2—best (i.e. maximum 2 mismatches, report best match). Mapping was performed in the following order: host rRNA; virus RNA; host RefSeq mRNA; host non-coding RNA. Reads which did not align to any of the aforementioned databases were then mapped to the host genome using STAR [39], again allowing a maximum of 2 mismatches per alignment.

To normalize for different library sizes, reads per million mapped reads (RPM) values were calculated using the sum of positive-sense virus RNA reads and host RefSeq mRNA reads as the denominator. For viral genome coverage plots, and for meta-analyses of host RefSeq mRNA coverage, mapping positions of RPFs were offset + 12 nt to approximate the location of the ribosomal P-site [5]. To calculate the length distributions of host- and virus-mapped RPFs, only those reads with a 5′ end + 12 nt offset mapping between the 16th nucleotide 3′ of the initiation codon and the 16th nt 5′ of the termination codon within coding regions (*gag* gene for virus; RefSeq mRNA coding regions for host) were counted. Histograms of the 5′ end positions (+ 12 nt offset) of host mRNA reads relative to initiation and termination codons were derived from reads mapping to RefSeq mRNAs with annotated coding regions ≥ 450 nt in length and with annotated 5′ and 3′ UTRs ≥ 60 nt in length. To estimate the *gag* stop codon readthrough efficiency, the expression levels (reads per kilobase per million mapped reads; RPKM) of the *gag* and *pol* ORFs were calculated by counting RPFs and RNASeq reads with 5′ end + 12 nt offset positions mapping within the respective coding regions, excluding 15 nt immediately upstream and downstream of the initiation and termination codons.

The short read length used for RNASeq (~ 28–34 nt) prevented a robust de novo analysis of MuLV splice variants from these data.

## Additional file

**Additional file 1: Table S1.** Library composition statistics. **Figure S1**. RPF distributions on host mRNAs. **Figure S2**. Length distributions of reads mapping to viral and host RNA. **Figure S3**. Alignment of the 5′ regions of gammaretroviral genomic sequences. **Figure S4**. Codon-based alignment of gammaretroviral *pol* genes downstream of the *env* splice acceptor.

## Abbreviations

MuLV: murine leukemia virus
uORF: upstream ORF
CHX: cycloheximide
HAR: harringtonine
