# Supplementary material for: Ribosome profiling of the retrovirus murine leukemia virus
Source: Retrovirology. 2018 Jan 22;15:10. doi: 10.1186/s12977-018-0394-5 (PMC5778647; doi:10.1186/s12977-018-0394-5)
Supplement: Supplementary file 1 — Additional file 1: Table S1. Library composition statistics. Figure S1. RPF distributions on host mRNAs. Figure S2. Length distributions of reads mapping to viral and host RNA. Figure S3. Alignment of the 5′ regions of gammaretroviral genomic sequences. Figure S4. Codon-based alignment of gammaretroviral pol genes downstream of the env splice acceptor. [file 12977_2018_394_MOESM1_ESM.pdf]

Table S1. Library composition statistics.

condition	protocol	repeat number	total reads	host rRNA	host mRNA	vRNA
MuLV-infected	RiboSeq-HAR	1	29,905,213	17,573,501	2,756,705	165,656
MuLV-infected	RiboSeq-CHX	1	62,356,362	33,006,799	10,040,974	582,520
MuLV-infected	RNASeq	1	99,527,314	1,482,962	1,282,214	154,635
Mock	RiboSeq-CHX	1	43,094,925	25,920,498	5,057,967	80
Mock	RNASeq	1	34,775,440	1,029,062	1,821,065	67
MuLV-infected	RiboSeq-CHX	2	11,330,744	4,218,443	1,065,369	55,629
MuLV-infected	RNASeq	2	17,356,077	175,706	310,572	60,000
Mock	RiboSeq-CHX	2	7,929,327	4,423,625	527,718	29
Mock	RNASeq	2	17,913,345	177,718	1,394,606	24

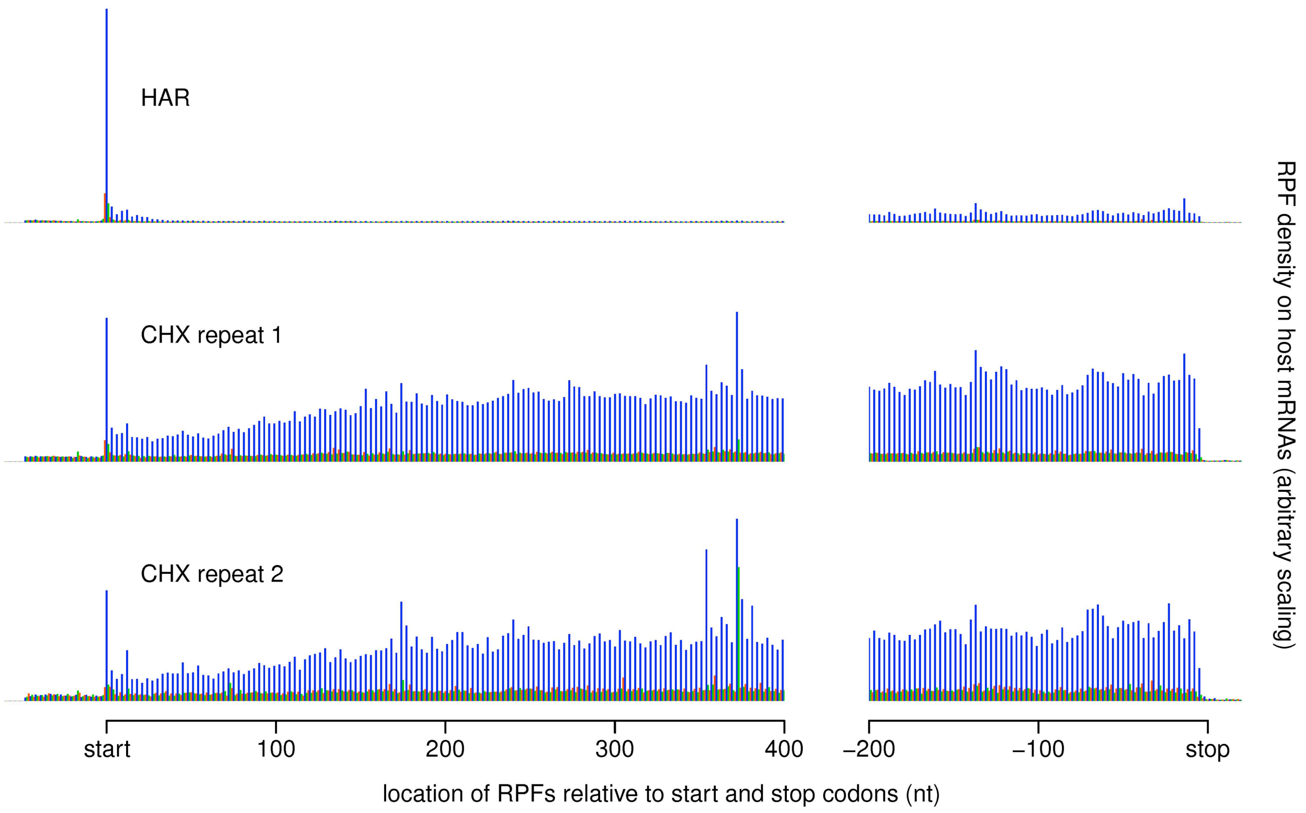

Figure S1. RPF distributions on host mRNAs. Histograms of RPF 5′ end positions with a +12 nt offset to approximate the ribosomal P-site, relative to annotated initiation and termination codons summed over all host RefSeq mRNAs for the RiboSeq libraries. Reads whose 5′ ends map to the first, second or third positions of codons are indicated in blue, green or orange, respectively. The majority of RPFs map to the first nucleotide of codons indicating excellent phasing of the RiboSeq libraries.

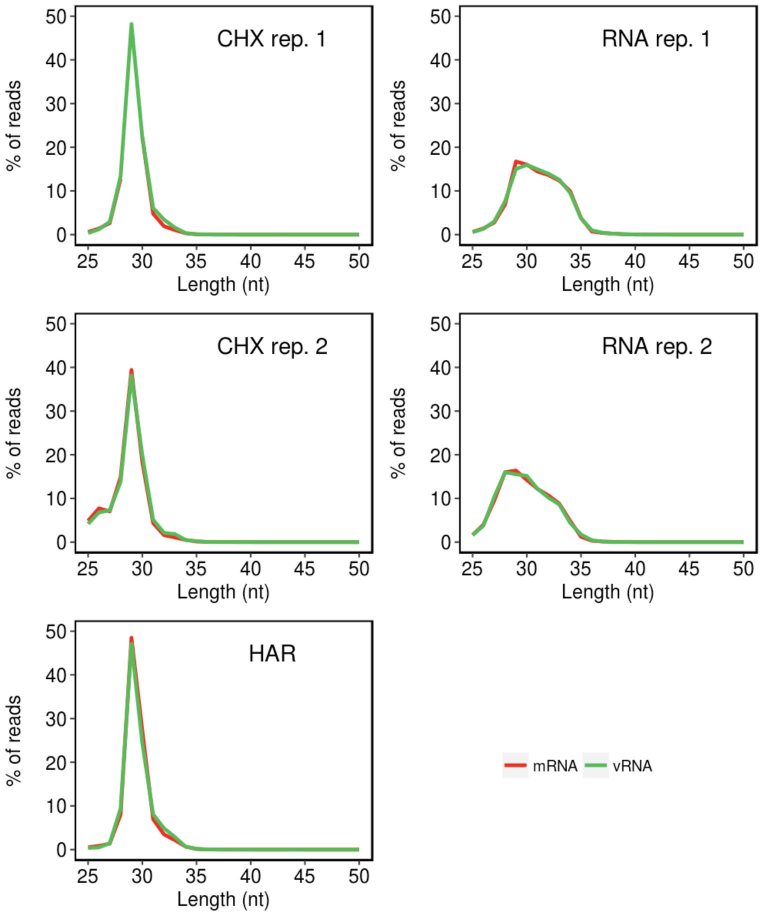

Figure S2. Length distributions of reads mapping to viral and host RNA. Line plots show the percentage of reads at the indicated length; green lines correspond to reads mapping within the viral *gag* ORF, and red lines correspond to reads mapping within the coding region of any host RefSeq mRNA. Close agreement between virus and host read-length distributions indicates absence of virus-related contamination [5].

```
AF033811    UAGCUCUGUAUCUGGCGGACCCGUG---GUGGAACUGACGAGUUC---GGAACACCCGGC    MoMULV
U94692      UAGCUCUGUAUCUGGCGGACCCGUG---GUGGAACUGACGAGUUC---GAGACACCCGGC    R-MULV
X02794      UAGCUCUGUACCUGGCGGACCCGUG---GUGGAACUGACGGGUUC---GAGACACCCGGC    F-MULV
J01998      UAGAUCUGUAUCUGGCGGCUCCGUG---GAAGAACUGACGAGUUC---GUAUU-CCCGAC    AKV-MULV
AF033812    UAGCUCUGUAUCUGGCGGACCCGUG---GUGGAACUGACGAGUUC---GGAACACCCGGC    A-MULV
AB060732    -----------------UGGAGUCG---AAGGAGCUGACGAGCUC---GAACUUCGCCCC    FeLV
KT724049    AAUCACUCUGUG--AGUGAUGUGAGUAGCGAACAGACGUGUUCGGGGGCUCACCGCCU    GaLV
KT724051    GAUCGCUCUGUG--AGCGACGUGUGAGUAGCGAGCAGACGUGUUCGGGGGCUCACCGCCC    WMSV
AB979448    GUCGGCCAACCUUCGUAGGAGUGUAGAGGGAGGACAGACGUGUCCUGGACC--CUCACAC    BaEV
KF786286    AAUCGAUCUAAG--AUCGAGGCGUGAGUAGCGGACAGACGUGUUCGGGGGCUCACCGCCC    KoRV
EF133960    UCUCAGACCGUAAGGACUGGAGGACUGUGAUCAGCAGACGUGCUAGGAGGAUCACAGG-C    PERV
JQ303225    GAGAAUUCUCACGGGAAGGAAAGUG---CGGGUUACGACCCUGGA---GGCUCCAUUACU    RfRv
AY842951    GAGGAUUUGGGAGGAUCGGAGUGGCG--------GGACGCUGCCGGGAAGCUCCACCUCC    REV
KF313137    GAGGGUUUGGGAGGAUCGGAGUGGCG--------GGACGCUGCCGGGAAGCUCCACCUCC    DIAV

AF033811    CGCAACCCUGGGAGACGUCCCAGGGACUUCGGGGGCCGUUUUUGUGGCCCGACCUGAGUC    MoMULV
U94692      CGCAACCCUGGGAGACGUCCCAGGGACUUCGGGGGCCAUUUUUGUGGCCCGGCCAGAGUC    R-MULV
X02794      CGCAACCCUGGGAGACGUCCCAAGGACUUCGGGGGCCAUUUUUGUGGCCCGGCCAGAGUC    F-MULV
J01998      CGCAGCCCUGGGAGACGUCUCAGAGGCAUCGGGGGCCCGCUGGGUGGCCCAAUCAGUAAG    AKV-MULV
AF033812    CGCAGCCCUGGGAGACGUCUCAGAGGCAUCGGGGGCCCGCUGGGUGGCCCAAUCAGUAAG    A-MULV
AB060732    CGCAACCCUGGAAGACGUUCCACGGGUGUCUGAUGUCUGGAGCCU-----------CUAG    FeLV
KT724049    GGUAAUCUUGGGAGACGUCCCAGGAUCAGGGGAGGACCAGGGACG---------------    GaLV
KT724051    GGUAAUCCUGGGAGACGUCCCAGGAUCAGGGGAGGACCAGGGACG---------------    WMSV
AB979448    GGUAAUCCUGGGAGACGUCCCAGGAUCAGGGGAGGACCAGGGACG---------------    BaEV
KF786286    GGCAAUCCUGGGAGACGUCCCAGGAUCAGGGGAGGACCAGGGACG---------------    KoRV
EF133960    UGCCGCCCUGGGGGACGCCCCGGGAGGUGGGGAGAGCCAGGGACG---------------    PERV
JQ303225    UGAAGUCGUAGGAGACGUCCCCGACGAGAAGGUGGCCCAGCGACG---------UCCGCA    RfRv
AY842951    GCUC--AGCAGGGGACGCCCUGAUCUGGGUUCUGUGGUAUCUGAU---------------    REV
KF313137    GCUC--AGCAGGGGACGCCCUGGCCUGAGUUCUGUGAUUUCUGAU---------------    DIAV
                      *   ****
```

Figure S3. Alignment of the 5′ regions of gammaretroviral genomic sequences. Alignment positions corresponding to the GUG codon in MuLV at which translational initiation was detected in RiboSeq HAR libraries, and the CUG codon in MuLV at which Glyco-Gag synthesis was previously reported to initiate, are highlighted in green and purple, respectively. Key: MoMuLV, Moloney murine leukemia virus; R-MuLV, Rauscher murine leukemia virus; F-MuLV, Friend murine leukemia virus; AKV-MuLV, AKV murine leukemia virus; A-MuLV, Abelson murine leukemia virus; FeLV, feline leukemia virus; GaLV, gibbon ape leukemia virus; WMSV, woolly monkey sarcoma virus; BaEV, baboon endogenous virus; KoRV, koala retrovirus; PERV, porcine endogenous retrovirus; RfRV, Rhinolophus ferrumequinum retrovirus; REV, reticuloendotheliosis virus; DIAV, duck infectious anemia virus.

```
AF033811   CUC CAA GCU CAC UUA CAG GCU CUC UAC UUA GUC CAG CAC GAA GUC UGG AGA CCU CUG GCG   MoMULV
U94692     CUC CAA GCU CAU UUA CAG GCA CUC UAC CUG GUC CAG CAC GAA GUC UGG AGA CCG CUG GCG   R-MULV
X02794     CUC CAA GCU CAU UUA CAG GCA CUC UAC CUG GUC CAG CAC GAA GUC UGG AGA CCG UUG GCG   F-MULV
J01998     CUC CAA GCU CAC UUA CAG GCC CUC CAA ACG GUG CAG CGA GAA AUU UGG AAA CCA CUG GCC   AKV-MULV
AB060732   AUG CAG GCA CAU UUA CGC GCC CUG CAG CUG AUC CAA GAG GAG AUC CAG AGA CCU CUA GCA   FeLV
KT724049   UUA UUU ACU CAU UUA AAG GCU UUA GAA GUU GUG AGG ACC CAG AUC UGG CAC CAG AUC AAG   GaLV
KT724051   UUA UUU ACU CAU UUA AAG GCU UUA GAA GUU GUG AGG ACC CAG AUC UGG GAC CAG AUC AAG   WMSV
AB979448   CUA CAG GCC CGG CUA AAA GGA CUA CAA GCA GUA CAG GCC CAA AUC UGG GCC CCC UUG GCA   BaEV
KF786286   CUA UUU ACC CAC UUG AAG GCU UUA GAG GUU GUG AGG ACU CAG AUC UGG GAC CAG AUA AAA   KoRV
EF133960   UUG UUC UCU AGG CUC AAG GCG CUC GAG UGG GUG AGG CAG CGA GCG UGG AAG CAG CUC CGG   PERV
JQ303225   UAU CUG GCC AGU UUA AAG GCC CUA CAA GGG GUC CAA CAU GAG AUA UGG CCC CUA GUG AGU   RfRv
AY842951   CUG CUU AAG UCC CUA CAG GCC CUC CAA GCU ACU AGG UCU CUG GCU CGG GCA ACG CUG CGC   REV
KF313137   UUG CUU AAG UCC CUA CAG GCC CUC CAA GCC ACU AGG UCU CUG GCU CGG GCA GCG CUG CGU   DIAV

AF033811   GCA GCC UAC CAA GAA CAA CUG GAC CGA CCG GUG GUA CCU CAC CCU UAC CGA GUC GGC GAC   MoMULV
U94692     GCA GCU UAC CAA GAA CAA CUA GAC CGG CCG GUA GUA CCU CAC CCU UUC CGA GUC GGU GAC   R-MULV
X02794     GCA GCU UAC CAA GAG CAA CUA GAC CGG CCG GUA GUA CCU CAC CCU UUC CGG GUC GGU GAC   F-MULV
J01998     GAG GCC UAC CGG GAC CAA CUA GAC CAA CCA GUG AUA CCA CAC CCC UUC CGG AUU GGA GAC   AKV-MULV
AB060732   GCA GCC UAC CGA GAA AAG CUC CAA ACC CCG GUC GUG CCG CAC CCC UUC AAA CCA GGA GAC   FeLV
KT724049   GAG GUA UAU AAG CCC GGU ACC GUG --- --- ACA AUC CCC CAC CCG UUC CAG GUC GGG GAU   GaLV
KT724051   GAG GUG UAC AAG CCC GGU ACC GUG --- --- GCA AUC CCC CAC CCG UUC CAG GUC GGG GAC   WMSV
AB979448   GAA CUG UAC CGG CCA GGA CAU UCG --- --- CAG ACC AGC CAC CCC UUC CAG GUG GGG GAC   BaEV
KF786286   GAA GCC UAC AGG CCU GGU ACU GUG --- --- GCC AUU CCC CAC CCG UUC CAG GUC GGU GAC   KoRV
EF133960   GAG GCC UAC UCA GGA GGA GAC UUG --- --- CAA GUU CCA CAU CGC UUC CAA GUU GGG GAU   PERV
JQ303225   GAG GCC UAC UCA GGA GGA GAC UUG --- --- CAA GUU CCA CAU CGC UUC CAA GUU GGG GAU   RfRv
AY842951   GAC CAA CUG CCC CAG AAA GAA GCU CAG CAA GAC CGU ACC CCA CUG UUC CAA CCU GGU GAC   REV
KF313137   GAC CAA CUG CCC CAG AAA GAA GCU CAG CAA GAC CGU ACC CCA CUG UUC CAA CCU GGU GAU   DIAV
```

Figure S4. Codon-based alignment of gammaretroviral *pol* genes downstream of the *env* splice acceptor. Alignment positions corresponding to the CUG codons of env-uORF-1 and env-uORF-2 in MuLV are highlighted in green and purple, respectively. Potential alternative initiation codons for translation of the C-terminal domain of the integrase protein are highlighted in cyan. Virus name abbreviations are given in the Figure S3 caption.
